# Genetic influence of a *STAU2* frameshift mutation and *RELN* regulatory elements on performance in Icelandic horses

**DOI:** 10.1038/s41598-025-95593-8

**Published:** 2025-04-04

**Authors:** Heiðrún Sigurðardóttir, Susanne Eriksson, Adnan Niazi, Marie Rhodin, Elsa Albertsdóttir, Thorvaldur Kristjansson, Gabriella Lindgren

**Affiliations:** 1 Department of Animal Biosciences, Swedish University of Agricultural Sciences, P.O. Box 7023, Uppsala, SE-75007 Sweden; 2 Faculty of Agricultural Sciences, Agricultural University of Iceland, Hvanneyri, Borgarbyggð, IS-311 Iceland; 3 Independent Researcher, Kópavogur, IS-203 Iceland; 4 The Icelandic Agricultural Advisory Centre, Höfðabakka 9, Reykjavik, IS-110 Iceland; 5 Center for Animal Breeding and Genetics, Department of Biosystems, KU Leuven, Kasteelpark Arenberg 30, Leuven, BE-3001 Belgium

**Keywords:** Equine, Gait quality, Trainability, Precocity, Whole-genome sequencing, Candidate causal variant, Computational biology and bioinformatics, Genetics, Molecular biology

## Abstract

Selection for performance in horse breeding benefits from precise genetic insights at a molecular level, but knowledge remains limited. This study used whole-genome sequences of 39 elite and non-elite Icelandic horses to identify candidate causal variants linked to previously identified haplotypes in the *STAU2* and *RELN* genes affecting pace and other gaits. A frameshift variant in linkage disequilibrium with the previously identified haplotypes in the *STAU2* gene (r^2^ = 0.85) was identified within a predicted *STAU2* transcript. This variant alters the amino acid sequence and introduces a premature stop codon but does not appear harmful or disease-causing and is potentially unique to equine biology. A large portion of the *RELN* haplotype overlapped with an H3K27me3 modification mark, suggesting a regulatory role of this region. Despite the small sample size, the *RELN* haplotype’s effects were validated for tölt, trot, and canter/gallop. Additionally, the *RELN* haplotype significantly influenced the age at which horses were presented for breeding field tests, indicating a potential role of the region in precocity and trainability. Functional experiments are needed to further investigate the regions’ influences on biological processes and their potential impact on horse performance.

**Supplementary Information:**

The online version contains supplementary material available at 10.1038/s41598-025-95593-8.

## Introduction

Elite horses, excelling in various sports disciplines, offer not only significant economic value to breeders and owners but also embody the pursuit of excellence, raising a key question: how can breeding be optimized for peak performance and overall success? Performance traits typically arise from intricate genetic mechanisms influenced by environmental factors. These genetic mechanisms involve the combined additive effects of numerous genes, possibly augmented by epistatic interactions^1,2^. Selection for performance can benefit from a good understanding of the genetic foundations of these traits. Moreover, insights derived from animal models like horses can unlock critical knowledge about similar genetic mechanisms in humans, advancing our understanding of gene function and biological processes. While recent research has highlighted key genes for performance traits in horses^3,4^, the detailed knowledge of mutations and potential regulatory elements remains largely unexplored. In this study, we take a significant step forward by using the Icelandic horse, renowned for its versatile gaits, as a model to enhance our understanding of the underlying molecular mechanisms of gait traits and performance.

The Icelandic horse is famous for its distinctive capacity to perform five gaits, among which the lateral gaits pace and tölt are considered the breed’s most valuable performance traits. Previous studies led to the discovery of the single base change in the *doublesex and mab-3 related transcription factor 3* (*DMRT3*) gene, commonly referred to as the ‘gait keeper’ mutation that significantly impacts the gaiting ability of Icelandic horses^4,5^. Furthermore, a genome-wide association study (GWAS) identified a quantitative trait locus (QTL) on ECA22 associated with conformation of the back and croup^6^. This QTL appeared to have considerable effects on the quality of the lateral gaits tölt and pace in Icelandic horses. In a more recent GWAS using high-density single nucleotide polymorphism (SNP) genotype data, three new QTLs were identified for pace, with possible effects on other gaits as well^7^. These three QTLs were estimated to account for about 13% of the phenotypic variance in pace and over a quarter of the variance together with the *DMRT3* variant. Two of these QTLs were located within known genes: *staufen double-stranded RNA binding protein 2* (*STAU2*) and *reelin* (*RELN*). Both these genes are expressed in neural tissue and are, therefore, likely to affect the development or the function of the locomotor network. A mutation in *STAU2* has been shown to reduce motor coordination but enhance motor learning abilities in mice^8^, suggesting that it may impair basic motor output while facilitating the learning of gaits. *RELN* expression has been observed in spinal cord development^9^, and several mutations in mice and rats are associated with the ‘reeler phenotype’ that manifests as abnormal locomotor coordination^10–14^. Additionally, the *RELN* gene has been shown to play a role in associative learning in response to environmental stimuli^15,16^ and is highly co-expressed with the dopamine D1 receptor (*Drd1*) gene in mice^17^, suggesting its importance in behaviour. The *RELN* gene is also essential for regulating the activity levels in neural circuits and stabilizing the balance between excitation and inhibition in neural circuits^18^. By maintaining this equilibrium, the *RELN* gene supports proper communication and function across neural networks, influencing processes like motor coordination, learning, and behaviour. Two common haplotypes have been identified for each of these two genes, all four of which appeared to affect pacing ability and gait quality in Icelandic horses. The most common haplotype in the *STAU2* gene was shown to have a large positive effect on pace quality and ability. In contrast, the two *RELN* haplotypes appeared to have opposing effects on pace quality and the quality of other gaits. Additionally, there were indications of genetic interactions between these novel genes and the *DMRT3* gene.

In the present study, whole-genome sequence (WGS) data for 39 carefully selected Icelandic horses was employed to explore a more complete set of variants within the previously identified haplotype regions (chr9:13,198,591 − 13,370,069 and chr4:4,222,615-4,228,503) within the *STAU2* and *RELN* genes, respectively^7^. The primary objective was to find candidate causal mutations responsible for the observed effects of these haplotypes on pace and other gaits and to enhance the annotations of the *STAU2* and *RELN* gene sequences.

## Results

### Variant calling

The average whole-genome depth of coverage of the sequenced samples ranged from 7.7-16.8x (~ 10.7x on average). The depth of coverage of the more specific regions of interest (*STAU2* and *RELN* gene) ranged from 6.9-15.6x (chr9:13,155,196 − 13,453,483 (*STAU2*)) and 6.8-15.2x (chr4:4,127,654-4,586,670 (*RELN*)), with an average of 9.5x for both regions.

### Linkage disequilibrium estimates

LD estimations, based on the top SNP (rs1147493591) in the *STAU2* gene identified in a previous GWAS^7^, revealed a haplotype containing 54 variants with an r² value higher than 0.8. The haplotype boundaries were identified at positions chr9:13,198,591 − 13,448,796, resulting in a haplotype length of 250 kb, which is approximately 80 kb longer than previously observed with SNP genotype data.

LD calculations based on the previously identified SNPs in the *RELN* gene^7^ revealed 24 variants in LD (r^2^ > 0.8) with the top SNP (rs69576243). The boundaries of this haplotype were the same two SNPs previously identified in the GWAS, so the length of the *RELN* haplotype remained unchanged at 5.9 kb (chr4:4,222,615-4,228,503).

## Variant analyses

### *STAU2* horse variants

**Fig. 1:**
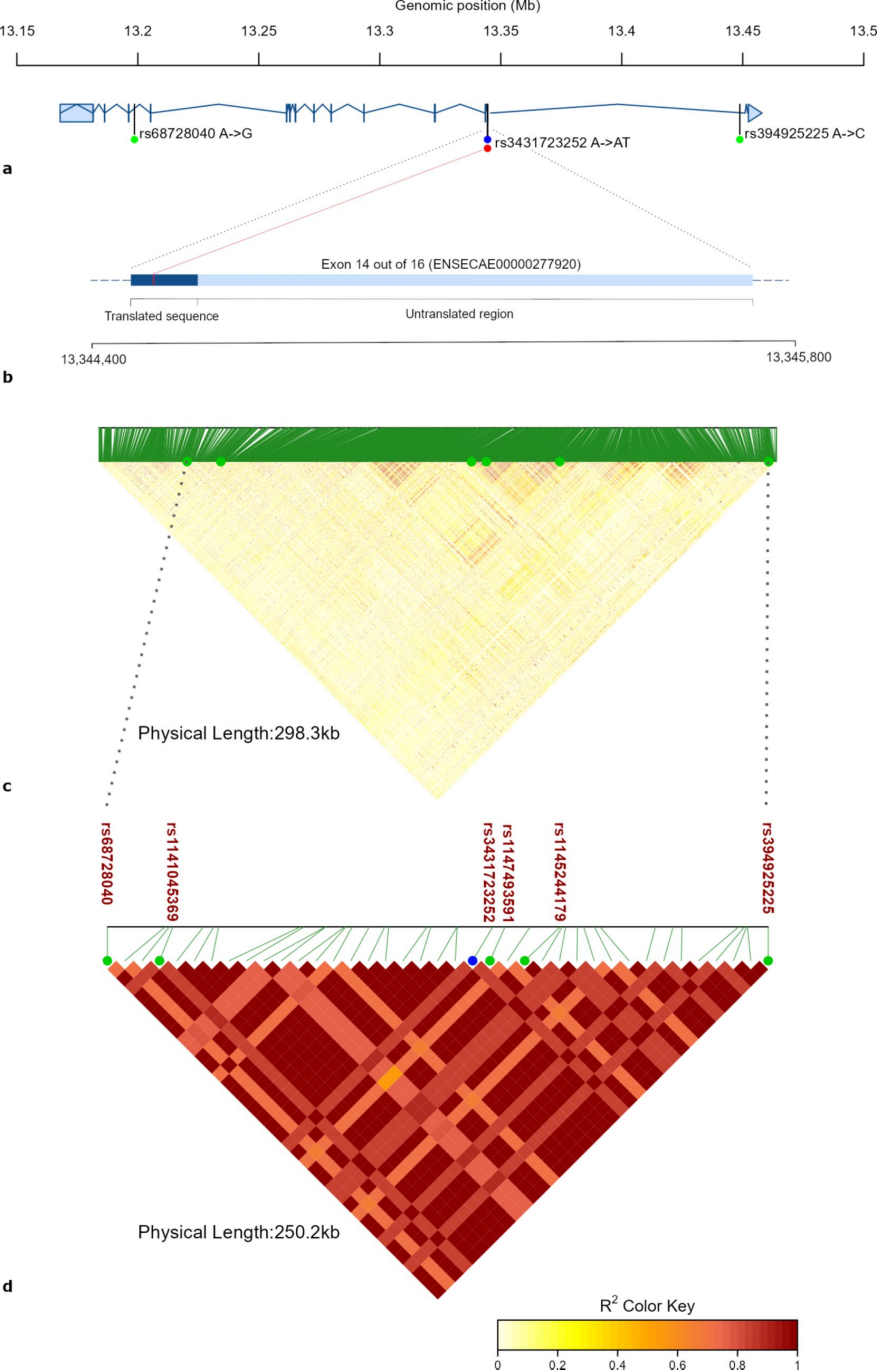
*STAU2* gene structure model and haplotype LD heatmaps. **(a)** Structure of the predicted *STAU2-206* transcript (chr9:13,167,860 − 13,452,304, ENSECAT00000063627.2, forward strand), showing the location of the identified frameshift variant (rs3431723252) within exon 14 out of 16. The haplotype boundaries are indicated by the positions of the first variant (rs68728040) and the last variant (rs394925225) in the haplotype. Exons are represented by blue vertical lines. **(b)** Detailed model of exon 14 (ENSECAE00000277920), highlighting the precise location of the frameshift variant within this exon. **(c)** A heatmap displaying the LD between all annotated variants within the horse *STAU2* gene (chr9:13,154,734–13,453,513). **(d)** A heatmap displaying the LD among the 39 variants within the *STAU2* haplotype region (chr9: 13,198,591 − 13,448,796). The variants shown in both heatmaps are the four SNPs previously identified with GWAS^7^ (rs68728040, rs1141045369, rs1147493591 and rs1145244179) of which rs68728040 marks the lower boundary of the haplotype. Additionally, the SNP marking the upper boundary of the haplotype (rs394925225) is shown, along with the frameshift variant rs3431723252 (marked with blue).

The horse *STAU2* gene, located at chr9:13,154,734–13,453,513, has eight predicted transcript variants (*STAU2-201* to *STAU2-208*) with lengths varying from 1,866 bp to 4,060 bp, each containing 10 to 16 exons. The refined *STAU2* haplotype encompasses a large part of all of these transcripts. VEP analysis for the haplotype revealed a frameshift variant (rs3431723252, NC_009152.3:g.13344526dup) in exon 14 (ENSECAE00000277920) out of 16 in the *STAU2-206* transcript (chr9:13,167,860 − 13,452,304, ENSECAT00000063627.2, forward strand) (Fig. 1a and b). The haplotype encompasses exons 5 to 14 in this transcript. The frameshift variant was predicted to have a high impact and to result in a change to the amino acid encoded (D/Dx). The locus had a GERP score of 0.05, indicating very weak evolutionary conservation, suggesting that this position is likely subject to little or no selective constraint across multiple species.

Further LD analysis based on the frameshift variant identified 39 variants in LD (r² > 0.8) with the same haplotype boundaries (chr9:13,198,591 − 13,448,796) (Fig. 1c and d) as the abovementioned haplotype based on the previously identified top SNP^7^. A list of all 39 variants comprising this *STAU2* haplotype is provided in Supplementary Table S1. Results from VEP analysis and GERP scores for the same list of variants are provided in Supplementary Table S2.

FAANG data^19,20^ revealed that one of the 39 variants (rs1149068012, chr9:13,303,604) overlapped with the histone H3 at lysine 4 monomethylation (H3K4me1) modification mark, which is commonly associated with gene enhancers^21–23^, in horse brain tissue (chr9:13,303,509 − 13,303,855) and adipose tissue (9:13,303,093 − 13,303,629). Another variant (rs396678054, chr9:13,440,620) also overlapped with this histone modification mark in horse ovary tissue (chr9:13,440,351 − 13,440,696). No variants overlapped with known histone modification marks in other available tissues.

### *RELN* horse variants

**Fig. 2:**
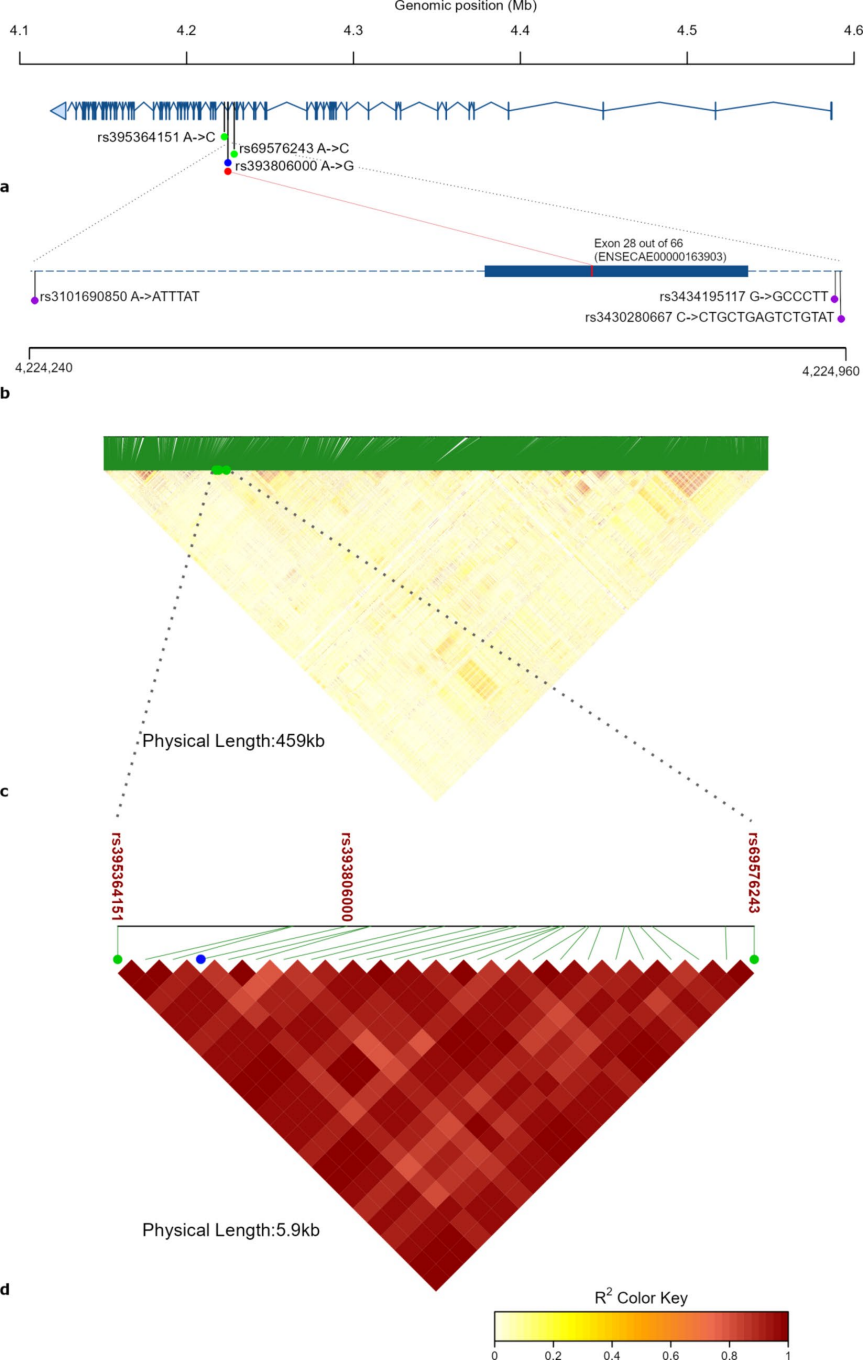
*RELN* gene structure model and haplotype LD heatmaps. **(a)** Structure of the Ensembl canonical *RELN* transcript (chr4:4,127,552-4,586,703, ENSECAT00000022978.4, reverse strand), showing the location of the identified synonymous variant (rs393806000) within exon 28 out of 66. The haplotype boundaries are indicated by the positions of the first variant (rs395364151) and the last variant (rs69576243) in the haplotype. Exons are represented by blue vertical lines. **(b)** A detailed model of exon 28 (ENSECAE00000163903) highlighting the exact location of the synonymous variant within this exon, along with the insertions (rs3101690850, rs3434195117, and rs3430280667) flanking it, all marked in purple. **(c)** A heatmap displaying the LD between all annotated variants within the horse *RELN* gene (chr4:4,127,552-4,586,703) **(d)** A heatmap showing the LD between the 24 variants comprising the *RELN* haplotype (chr4:4,222,615- 4,228,503). The variants shown in both heatmaps were the two SNPs identified by the previous GWAS^7^ (rs395364151 and rs69576243) which were also the boundaries of the refined haplotype, along with the synonymous variant identified in exon 28 (marked with blue).

The horse *RELN* gene, located at chr4:4,127,552-4,586,703, has five predicted transcript variants (*RELN-201* to *RELN-205*), of which three are inferred from homology (*RELN*-203 to *RELN*-205). The length of the five transcripts varies from 11,119 bp to 22,583 bp, each containing 63 to 66 exons. The refined *RELN* haplotype encompasses only exon 28 (ENSECAE00000163903). Within the *RELN* haplotype (Fig. 2), the VEP analysis identified one synonymous exonic variant (rs393806000, NC_009147.3:g.4224738 A > G) that, by definition, does not alter the encoded amino acid. Additionally, small insertions were found on either side of exon 28: rs3101690850 (NC_009147.3:g.4224244_4224245insTTTTA), rs3434195117 (NC_009147.3:g.4224956_ 4224957insCCCTT) and rs3430280667 (NC_009147.3:g.4224959_ 4224960insGCTGAGTCTGTATT). GERP scores indicate that the insertion loci are within weak to moderate evolutionary conservation (0.89–2.72), suggesting that these sites may be under relaxed selective pressure up to some level of purifying selection. The synonymous variant locus had a GERP score of -2.48, indicating that it is within a region of low evolutionary constraint, suggesting potential neutrality or weak negative selection at this position. A complete list of variants comprising the *RELN* haplotype is provided in Supplementary Table S3. Results from the VEP analysis and GERP scores for the same list of variants are provided in Supplementary Table S4.

FAANG data^19,20^ revealed that over half of the variants in the *RELN* haplotype (chr4:4,226,638- 4,228,503) overlapped a wide histone H3 at lysine 27 trimethylation (H3K27me3) modification mark (chr4:4,226,401-4,237,199) in equine brain tissue. No variants overlapped with known histone modification marks in other available tissues.

### Human translated variants

Translated genomic coordinates of the two haplotypes from EquCab3.0 into human (GRCh38/hg38) genomic coordinate identities were chr8:73,424,997 − 73,677,306 (*STAU2* haplotype) and chr7:103,582,019–103,591,772 (*RELN* haplotype). According to VEP analysis, none of the translated *STAU2* human variants were exonic. However, the group did include several regulatory variants (enhancers and CTCF binding sites) as well as some variants predicted to be associated with nonsense-mediated mRNA decay (NMD) regulation. VEP analysis for the translated human variants revealed that the exonic variant in the horse *RELN* haplotype identifies with a missense variant in the human genome that results in a change in amino acid encoding (asparagine (N) to lysine (K)). This change was predicted to have deleterious protein function effects (SIFT = 0.01–0.02). However, according to PhyloPhen scoring, it was predicted to have benign effects (0.01–0.031). This human missense variant was observed to be somewhat conserved across taxa according to PhyloP scores (0.41–2.14) based on different species sets and alignment algorithms. Furthermore, several variants predicted to be associated with NMD regulation were identified among the translated human *RELN* variants. The insertions identified on either side of the identified exon in the horse genome were not annotated in the human genome. Results from VEP analysis, GERP and PhyloP scores are provided for the complete list of translated *STAU2* and human *RELN* variants in Supplementary Table S5 and Table S6, respectively.

Further investigation into regulatory elements revealed that several translated variants of the *STAU2* and *RELN* genes overlapped with predicted transcription factor binding sites. According to the GeneHancer database^24^, the *STAU2* gene is known to be targeted by ten of the identified transcription factors (FOS, ATF7, JUNB, MEF2D, THRB, Pou5f1, ZNF384, TEAD3, ZSCAN4, and KLF9), while the *RELN* gene is targeted by three of the identified transcription factors (Mafk, ZBTB26, and HMBOX1) (see Supplementary Table S7 and S8). None of the translated variants were associated with known changes in gene expression levels according to the GTEx portal. However, within the region of the *STAU2* variants, other variants were associated with eQTL in nerve tissue (tibial) and sQTL in muscle-skeletal tissue, among different tissue types. Similarly, within the region of the translated *RELN* variants, other variants were associated with sQTL in nerve tissue (tibial) and eQTL in heart tissues (atrial appendage and left ventricle). Additionally, a few variants within the *RELN* region were predicted with a high probability (84–99%) to act as splice sites in their alternative allele form, although these were not the translated variants. The same analysis for the *STAU2* region yielded no high-probability splice site predictions.

### Protein modelling

**Fig. 3:**
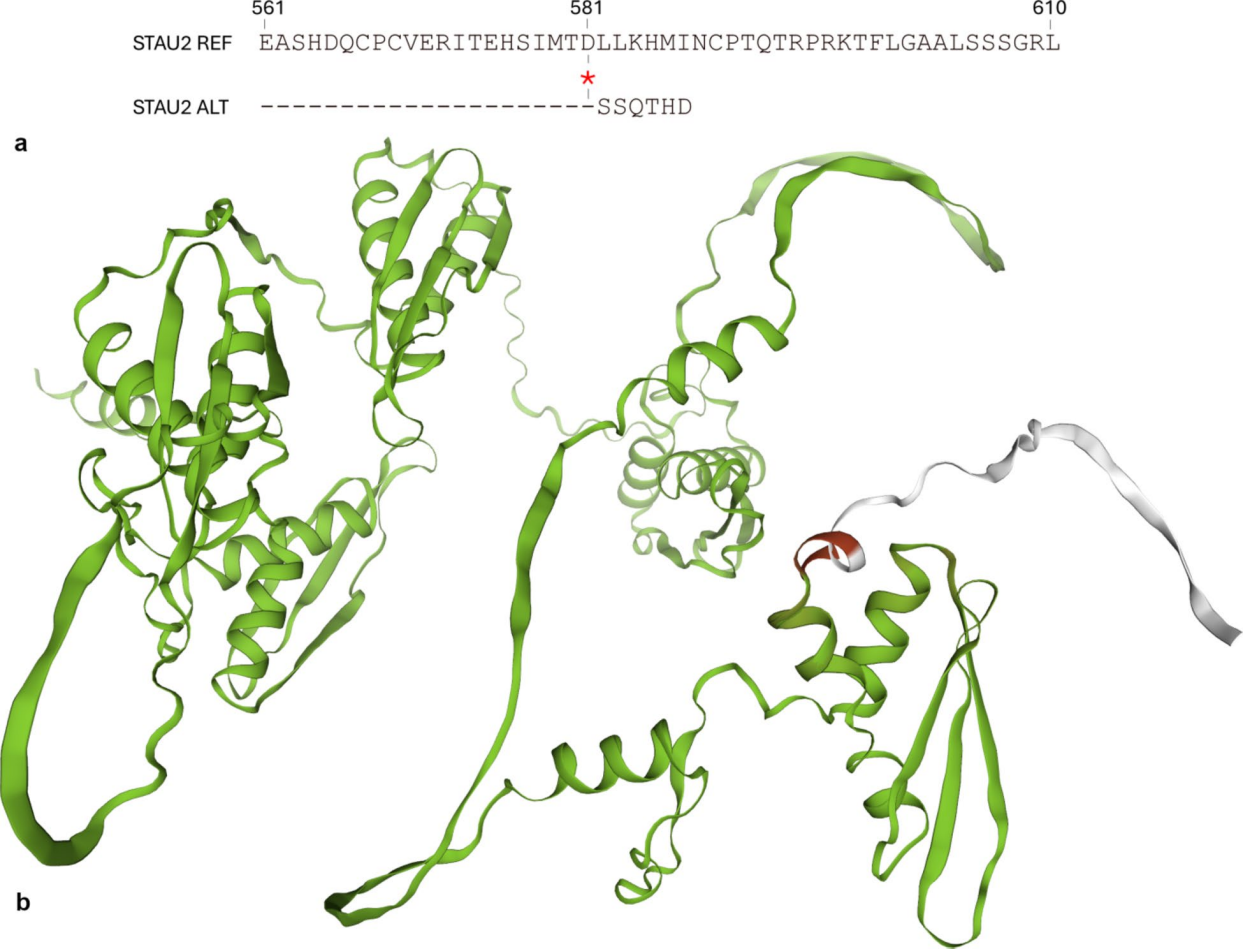
*STAU2* protein sequence and structure model. **(a)** Partial protein alignment of the predicted *STAU2* protein in horses carrying the reference haplotype (REF) and those carrying the alternative (ALT) haplotype. The location of the frameshift variant is highlighted with red asterisks, and dashes stand for sequence identities. **(b)** Protein modelling of consistency between the reference and the alternative *STAU2* horse protein. The green colour indicates complete consistency, the red indicates an inconsistency in encoded amino acids, and the silver suggests the absence of encoded amino acids in the alternative protein.

Further investigation into the frameshift variant in the *STAU2* horse haplotype revealed that the mutation caused a change in amino acid encoding and a premature stop codon, reducing the encoded protein by approximately 4% (Fig. 3).

### Phenotypic differences between refined haplotypes

As previously observed^7^, the frequency of the *STAU2* reference haplotype is high within the group of Icelandic horses presented at breeding field tests. In the current sample, 29 horses (74%) were homozygous for the *STAU2* reference haplotype, while no horse was homozygous for the *STAU2* alternative haplotype. Ten horses (26%) were heterozygous carriers. The largest difference among the traits investigated between the homozygous and heterozygous horses was observed for age at assessment (*p* = 0.19), but neither this difference nor that for any of the other traits of interest was significant (*p* > 0.05) (Table 1).

**Table 1:** Phenotypic differences between individuals in the sequenced sample with two copies of the *STAU2* reference haplotype (SS genotype) and those heterozygous for the *STAU2* haplotype (Ss genotype). Differences were estimated using a general linear model with sex as a fixed effect. The table presents the number of observations (N), minimum (min) and maximum (max) phenotypic scores, least squares means (lsmeans) with standard errors (se), and *p*-values for lsmean differences between the SS and Ss genotypes. Age at assessment was recorded in years. Traits *Tölt*, *Slow tölt*,* Trot*,* Pace*,* Gallop*,* Canter*, and *Back and croup* were scored subjectively on a scale from 5.0 to 10. The trait *Pace ≥ 5.0* includes all Pace scores, including 5.0 (indicating the trait was not shown), whereas *Pace > 5.0* includes only scores for which the trait was shown. Morphological traits, including *Height at front* and *Backline*, were measured in centimeters. *Height at front* is defined as the difference between the highest point on the withers (M1) and the tuber sacrale (M3), while *Backline* is defined as the difference between the height at the tuber sacrale (M3) and the lowest point of the back (M2).

	Hom ref hap (SS)					Het ref/alt hap (Ss)					*p*-value
	*N*	min	max	lsmeans	se	*N*	min	max	lsmeans	se	
Age at assessment	29	4	14	7.4	0.9	10	6	7	6.2	0.8	0.192
Tölt	29	6.5	10.0	8.6	0.4	10	7.0	9.5	8.8	0.4	0.573
Slow tölt	29	6.0	10.0	8.3	0.4	10	6.5	9.5	8.4	0.3	0.614
Trot	29	6.0	9.5	8.3	0.3	10	6.5	9.5	8.6	0.3	0.399
Pace ≥ 5.0	29	5.0	9.5	7.0	0.6	10	5.0	9.0	7.0	0.5	0.954
Pace > 5.0	18	6.0	9.5	7.9	0.6	6	6.5	9.0	8.1	0.5	0.695
Gallop	29	7.0	9.5	8.3	0.2	10	7.5	9.0	8.3	0.2	0.860
Canter	29	6.5	9.5	8.1	0.3	10	6.5	9.0	8.2	0.3	0.744
Back and croup	29	6.5	9.5	8.4	0.3	10	7.0	9.5	8.7	0.3	0.368
Height at front (M1-M3)	29	1	9	5.9	0.8	10	2	10	6.8	0.8	0.285
Backline (M3-M2)	29	2	10	5.5	0.8	10	3	9	4.7	0.7	0.302

Among the 39 horses sampled, nine (23%) were homozygous for the *RELN* reference haplotype, and 14 (36%) were homozygous for the *RELN* alternative haplotype. Comparing the phenotypes of these two groups revealed significant differences (*p* < 0.05) in all traits except for pace and height at front (Table 2). Horses homozygous for the *RELN* reference haplotype had higher scores (*p* < 0.05) for back and croup conformation, tölt, slow tölt, trot, gallop, and canter. Additionally, they appeared to have a more balanced backline compared to those homozygous for the alternative haplotype (*p* = 0.04). Furthermore, horses with the reference haplotype were significantly younger at the time of their breeding field test than those with the alternative haplotype (*p* = 0.03). The results for tölt, slow tölt, trot, gallop, and canter are consistent with findings from a previous GWAS^7^, although that study also found significant differences for pace, with horses homozygous for the alternative haplotype scoring higher.

**Table 2:** Phenotypic differences between individuals in the sequenced sample with two copies of the *RELN* reference haplotype (rr genotype) and those with two copies of the *RELN* alternative haplotype (RR genotype). Differences were estimated using a general linear model with sex as a fixed effect. The table presents the number of observations (N), minimum (min) and maximum (max) phenotypic scores, least squares means (lsmeans) with standard errors (se), and *p*-values for lsmean differences between the rr and RR genotypes. Age at assessment was recorded in years. Traits *Tölt*, *Slow tölt*,* Trot*,* Pace*,* Gallop*,* Canter*, and *Back and croup* were scored subjectively on a scale from 5.0 to 10. The trait *Pace ≥ 5.0* includes all Pace scores, including 5.0 (indicating the trait was not shown), whereas *Pace > 5.0* includes only scores for which the trait was shown. Morphological traits, including *Height at front* and *Backline*, were measured in centimeters. *Height at front* is defined as the difference between the highest point on the withers (M1) and the tuber sacrale (M3), while *Backline* is defined as the difference between the height at the tuber sacrale (M3) and the lowest point of the back (M2).

	Hom ref hap (rr)					Hom alt hap (RR)					*p*-value
	*N*	min	max	lsmeans	se	*N*	min	max	lsmeans	se	
Age at assessment	9	5	7	5.9	0.9	14	5	14	8.3	1.0	0.029
Tölt	9	8.5	9.5	9.4	0.3	14	6.5	10.0	8.3	0.4	0.014
Slow tölt	9	7.5	9.5	8.9	0.4	14	6.5	9.5	8.0	0.4	0.049
Trot	9	8.0	9.5	9.1	0.3	14	6.0	8.5	7.8	0.3	< 0.001
Pace ≥ 5.0	9	5.0	9.0	7.3	0.6	14	5.0	9.0	7.1	0.7	0.776
Pace > 5.0	5	7.0	9.0	8.6	0.5	10	6.5	9.0	7.7	0.6	0.163
Gallop	9	7.5	9.5	8.7	0.2	14	7.0	8.5	8.0	0.2	0.010
Canter	9	7.0	9.0	8.4	0.3	14	6.5	9.0	7.8	0.3	0.040
Back and croup	9	8.0	9.5	9.2	0.3	14	6.5	9.5	8.2	0.4	0.008
Height at front (M1-M3)	9	4	9	7.3	0.8	14	1	10	5.5	1.0	0.072
Backline (M3-M2)	9	3	8	4.5	0.7	14	3	10	6.3	0.8	0.041

### Allele frequencies

Allele frequency estimates for the *STAU2* frameshift variant, the top GWAS SNPs in the *STAU2* and *RELN* haplotypes, and the gait-keeper mutation in the *DMRT3* gene are shown in Table 3 for Icelandic horses from both this study (39 horses) and the previous GWAS (379 horses)^7^. The allele frequencies were similar across both sample groups. Based on publicly available data^25–31^, estimates for the same alleles in other gaited and non-gaited breeds are provided in Supplementary Table S9. In all the breeds, the *STAU2* frameshift variant and the top *STAU2* SNP display a similar pattern as in the Icelandic horse, with nearly identical estimates for both variants and a notably higher frequency of the reference allele. No apparent differences in allele frequencies were observed between gaited and non-gaited breeds. In contrast, the *RELN* variant showed greater variability in allele frequencies across breeds, though no distinct patterns related to gaiting abilities were evident. Generally, the alternative allele was, however, more frequent (C > 0.50).

**Table 3:** Allele frequencies of the frameshift variant in the *STAU2* haplotype, the top SNPs in the *STAU2* and *RELN* haplotypes, and the *DMRT3* ‘gait-keeper’ mutation in Icelandic horses from this study, shown for the total sample and for elite and non-elite groups, with comparison to a previous GWAS^7^. The table presents the number (N) of horses and the frequency of the reference (ref) and alternative (alt) alleles. Frequencies for the same alleles in other gaited and non-gaited breeds are in supplementary table S9.

	*N*	*STAU2*		*STAU2*		*RELN*		*DMRT3*	
		Frameshift variant		Top SNP		Top SNP		Gait keeper	
		chr9:13,344,525		chr9:13,349,141		chr4:4,228,503		chr23:22,391,254	
		ref	alt	ref	alt	ref	alt	ref	alt
		A	AT	A	G	A	C	C	A
Current study: Total sample	39	0.88	0.12	0.88	0.12	0.44	0.56	0.05	0.95
Current study: Elite group	22	0.86	0.14	0.84	0.16	0.59	0.41	0.00	1.00
Current study: Non-elite gr.	17	0.91	0.09	0.94	0.06	0.24	0.76	0.12	0.88
Previous GWAS	379	−	−	0.90	0.10	0.45	0.55	0.05	0.95

## Discussion

In this study, we investigated specific loci associated with gaits in Icelandic horses, building on previous GWAS findings^7^, with the goal of identifying candidate causative variants for these equine traits. This was achieved through fine-mapping of critical genomic regions in a precisely selected cohort of horses exhibiting a range of relevant phenotypes.

The identified *STAU2* frameshift variant leads to an alteration in the amino acid sequence and introduces a premature stop codon, severely impacting the production of the *STAU2* protein. Remarkably, despite this substantial change, the variant does not appear to be harmful or disease-causing in horses. Its minimal evolutionary constraint suggests it is not conserved across species, and it seems to be absent from both the human (hg38) and mouse (mm39) genomes.

The *STAU2* gene encodes a double-stranded RNA-binding protein that plays a role in mRNA transport^32^ and contributes to the asymmetric distribution of mRNA in dividing intermediate progenitor cells^33^, a process that influences neuron proliferation and fate determination. Studies in mice have shown that the loss of *STAU2* affects neuron survival, resulting in impaired motor coordination but enhanced motor learning abilities^8^.

Our previous GWAS^7^, involving 362 horses with breeding field test scores, revealed a significant difference in pace quality between horses with two copies of the *STAU2* reference haplotype and those that were heterozygous or carried two copies of the alternative haplotype. Notably, horses with higher test scores for trot and gallop tended to carry one or two copies of the alternative haplotype, while those with higher pace scores carried two copies of the reference haplotype. The identification of the frameshift variant as the likely cause of the suggested differences in gait quality observed in our previous GWAS^7^, implies that the insertion causing the frameshift is disadvantageous to pace but beneficial for trot and gallop. However, this pattern was not as clear in our current dataset of 39 horses, likely due to the small sample size and the high prevalence of the reference haplotype. The Icelandic horses included in this study were selected solely based on phenotypes and not genotypes. The imbalance in haplotype frequency is thus understandable, given the generally low frequency of the alternative allele across the breed, observed in our previous GWAS^7^. The high frequency of the reference allele at the *STAU2* locus in Icelandic horses may be attributed to its strong positive effect on pace quality, which is a highly valued trait in Icelandic horse breeding^34^. However, the reference allele is also common in other breeds (A ≥ 0.63), regardless of gaiting ability. This strongly indicates that the effects of the frameshift variant are not specific to lateral gaits such as pace but instead have a more general role in the performance of horses.

It is also plausible that the reference haplotype, previously seen to positively impact pace, is associated with motor learning abilities, as seen in mice^8^, and may interact with the *DMRT3* gait keeper variant, as observed in our previous GWAS^7^. The *DMRT3* variant typically plays a dominant role in gaiting ability, with one copy of the mutant allele (CA genotype) enhancing the horse’s natural tölt ability and two copies (AA genotype) enabling the development of pace^4,5^. In our previous study^7^, we found that horses with the CA genotype at the *DMRT3* locus had the highest prevalence of the *STAU2* reference haplotype, and some even received pace scores despite the lack of the AA genotype in the *DMRT3* gene. This led to the hypothesis that the reference haplotype might compensate for the absence of the mutant allele (A) at the *DMRT3* locus. However, we could not confirm this in the current study due to the small sample size, which included only four CA horses, all classified as non-elite. Still, the pattern persists: all the horses with CA genotype in the *DMRT3* locus in this study were homozygous for the reference haplotype, and one of them received a pace score of 6.5, meaning that it showed pace.

Given the ambiguity surrounding the role of the *STAU2* frameshift variant as well as the merely predicted existence of the horse *STAU2*-206 transcript, we strongly recommend conducting further functional experiments. These studies will help elucidate the variant’s precise function, its influence on biological processes, and its potential impact on horse performance.

The refined horse *RELN* haplotype contained a single synonymous variant within the exon covered by the haplotype, along with 5–14 bp insertions located both upstream and downstream of this exon. These variants may affect gene regulation, potentially altering protein expression, structure, and function, which could explain the significant phenotypic effects linked to the haplotype. Additionally, a large portion of the haplotype overlaps with a wide H3K27me3 modification mark, which is typically associated with gene downregulation^21,35^. This indicates that the haplotype may play a role in regulating the expression of the *RELN* gene, or other genes in the vicinity. Several human *RELN* variants were also predicted to be associated with nonsense-mediated mRNA decay (NMD) regulation, further highlighting the region’s regulatory significance.

The synonymous variant identified in the horse corresponds to a missense variant in humans, which may be disease-causing. Mutations in the human *RELN* gene have been linked to Lissencephaly 2 (Norman-Roberts type)^36^ and familial temporal lobe epilepsy-7 (ETL7)^37^. The *RELN* gene encodes an extracellular matrix protein that is essential for cell-to-cell interactions during neuronal development, particularly for the migration and positioning of neurons^38–41^. Loss of the reelin protein can prevent neurons from positioning correctly, leading to severe brain defects and neurological disorders such as schizophrenia, autism, bipolar disorder, and major depression in humans^42–44^. However, there is no evidence to suggest that either the reference or the alternative *RELN* haplotype contributes to severe phenotypes in horses. Instead, our results support previous observations of an association with performance^7^. Specifically, we observed significant differences in gait performance between horses homozygous for the reference haplotype and those homozygous for the alternate haplotype across all traits studied except pace. A possible explanation for the non-significant association with pace in this study, as opposed to the significant association in the previous study^7^, is the division of the already small sample into smaller groups of four-gaited (*N* = 15) and five-gaited (*N* = 24) horses. Additionally, all horses homozygous for the *RELN* reference haplotype – previously shown to be more common in four-gaited horses^7^ – were elite, while the majority of horses homozygous for the alternative haplotype – previously linked to better pace performance^7^ – were non-elite. This likely contributed to the non-significant result for pace, which was somewhat expected, as the objective for the small dataset was not to examine phenotypic differences.

All horses in the sample were selected based solely on their phenotypes, without prior knowledge of their genotypes before sequencing. Interestingly, all horses homozygous for the reference *RELN* haplotype were elite horses. The distinction between elite and non-elite horses was determined using standardized breeding field test scores in accordance with FEIF regulations, ensuring consistency across countries. Therefore, the imbalance in the origin of the horses can be assumed to have a negligible influence on the classification into elite and non-elite groups. The horses homozygous for the reference *RELN* haplotype were also significantly younger at the time of their breeding assessments compared to horses homozygous for the alternative haplotype. However, this age difference may partly be attributed to the elite horses’ favourable conformation, specifically in height at the front and backline, which has been shown to influence riding ability and the age at which horses are first presented at breeding field tests^45^. While the *RELN* haplotype may not fully account for the observed age difference, its influence cannot be entirely excluded. The age difference, together with the positive effect of the reference haplotype across gaits, may suggest a broader benefit for overall performance rather than specific movement patterns or gait attributes. Supporting this, the *RELN* gene has been shown to play a role in synaptic plasticity and in regulating DNA methylation in the adult central nervous system of rats, particularly in the hippocampus during associative learning in response to environmental stimuli^15,16^. Given the potential role of the *RELN* haplotype in regulating *RELN* gene expression, there could be a link to trainability or precocity in horses, where associative learning plays a key role^46–48^. Additionally, *RELN* is highly co-expressed with the dopamine D1 receptor (*Drd1*) gene in mice^17^, while in horses, a related gene – the dopamine D4 receptor (*DRD4*) – has been associated with temperament^49^. The *RELN* gene is also essential for fine-tuning GABAergic connectivity, the network of inhibitory synapses that modulate brain activity and maintain the balance between excitation and inhibition in neural circuits^18^. Enhanced trainability and precocity, which may make Icelandic horses more likely to be ready for breeding assessments at a younger age, are valuable traits in breeding. However, given the difficulty in distinguishing between the direct effects of the *RELN* haplotype and the indirect effects of conformation – along with previous GWAS findings indicating a more locomotion-specific effect^7^ – further research is needed to explore *RELN*’s regulatory role in precocity and trainability. Additionally, further investigation of allele frequencies in other breeds with larger sample sizes could help establish patterns of effects.

In conclusion, a likely candidate variant responsible for the *STAU2* haplotype effect on pace, identified in a previous GWAS, was discovered. This single-base frameshift mutation alters the amino acid sequence and introduces a premature stop codon. Additionally, a significant portion of the *RELN* haplotype overlaps with a histone modification mark, suggesting a regulatory role potentially linked to precocity. This study has enhanced our understanding of the genetic foundation of gaits and performance in the Icelandic horse significantly. However, functional experiments are needed to confirm these findings and validate the phenotypic effects of these genetic elements.

### Methods

#### Selection of horses

The horses used for this study were carefully selected based on their lateral gait performance at standardized breeding field tests^34^. The phenotype data used for the selection process were retrieved from the Worldfengur database^50^, and the main selection criteria were breeding field test scores for the gaits tölt and pace and the conformation of back and croup, along with morphological measurements for back inclination and height at the front. These conformation and measurement traits were considered because they have been shown to significantly affect lateral gait performance in Icelandic horses^6,45^.

### Phenotype descriptions

The gait traits tölt and pace are both considered lateral gaits. Tölt is a symmetrical four-beat ambling gait with an ipsilateral sequence of footfall and a large speed variation but without a suspension phase. In contrast, pace is ridden at high speed and described as a symmetrical, two-beat gait in which ipsilateral legs move nearly synchronously back and forth with a visible suspension phase. Features of both gaits, such as beat, suppleness, stride length, leg-action, speed capacity, collection and lightness, are considered when being assessed^34^. In addition to tölt and pace, breeding field test scores for the gait traits trot, canter and gallop were used for this study. Trot is a symmetrical two-beat, diagonal gait with a moment of suspension, whereas both canter and gallop are asymmetrical gaits with a suspension phase. Canter is characterised as a three-beat, medium-speed gait, whereas gallop is a four-beat, high-speed gait^34^.

**Fig. 4:**
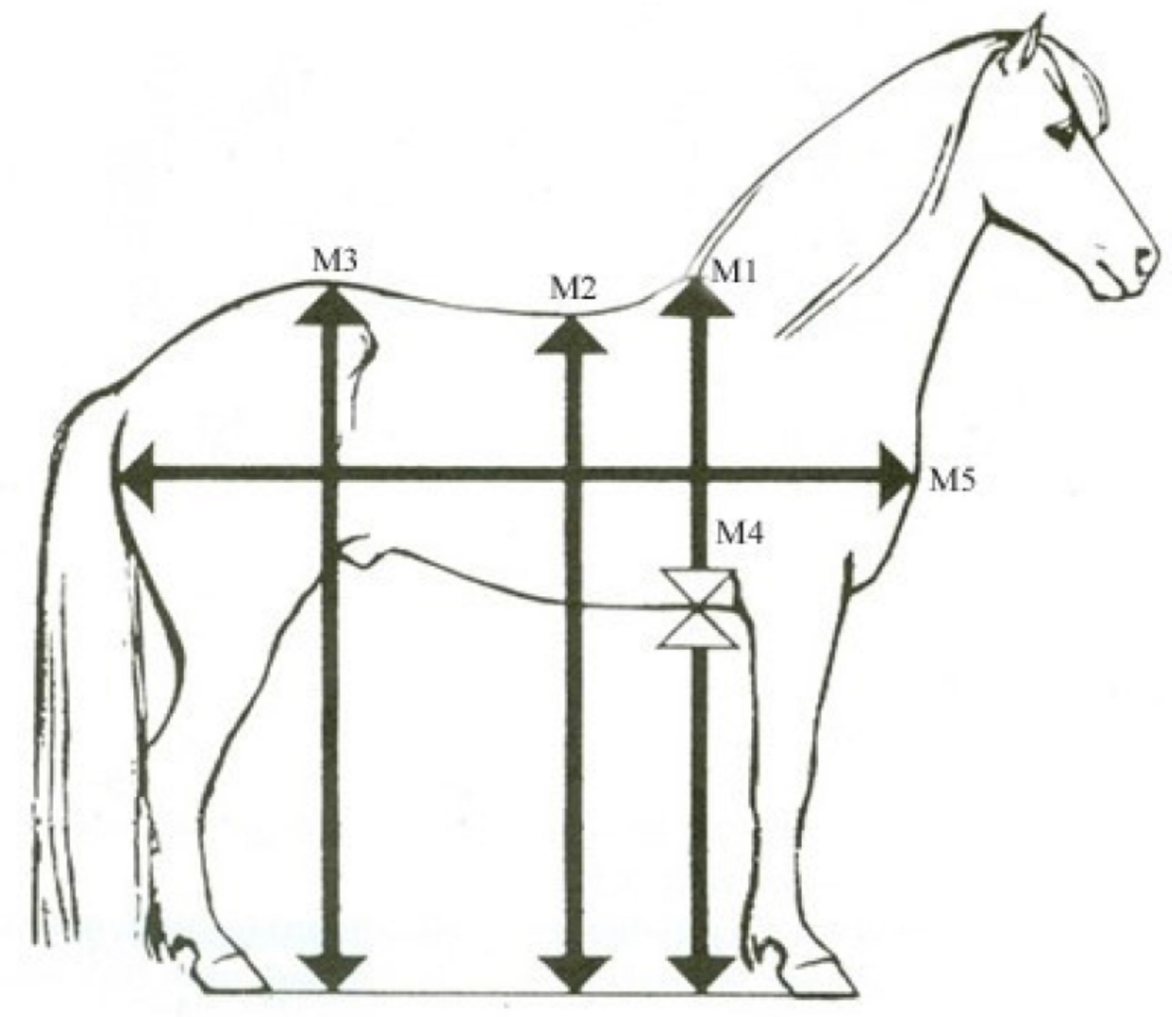
Body measurements recorded during breeding field tests. A figure showing some morphological traits routinely measured with a rod at standardized breeding field tests^51^. Original image created by Pétur Behrens. The height at the front is the difference between the highest point on withers and the highest point of the tuber sacrale (M1-M3). The back inclination is defined as the difference between the highest point of the tuber sacrale and the lowest point on the back (M3-M2).

The standardized assessment of the back and croup involves examining several key features, whereof the shape and inclination of the backline (the line extending from the base of the withers to the tuber sacrale) is the most important one. Other features assessed include the width and muscularity of the back, the length and width of the loin (the area covering the lumbar vertebrae, extending from the last rib to the sacrum), and the croup’s length, slope, shape, and muscularity^34^. The morphological trait back inclination is defined as the difference between the height at the tuber sacrale (M3) and the lowest point of the back (M2), and the height at front is defined as the difference between the highest point on withers (M1) and the tuber sacrale (M3) (Fig. 4).

The gait traits and back and croup conformation were subjectively scored at standardized breeding field tests on a scale from 5.0 to 10.0, with 0.5 intervals, based on a judging scale established by the International Federation of Icelandic Horse Associations, FEIF^34^, while the morphological traits were measured in centimetres with a rod. According to the FEIF judging scale, a score of 5.0 indicates that a trait was not demonstrated during the test, while a score of 9.5–10.0 represents the breeding goal for that trait^34^. The subjective assessments are conducted by a panel of three internationally certified judges who are routinely rotated between standardized breeding field tests both within and between countries to ensure consistency and comparability of assessments across tests.

### Elite horses vs. non-elite horses

The selected horses were categorised into elite and non-elite horses by three trained breeding judges, based on prior breeding field assessments of the relevant traits and the test score criteria outlined in Supplementary Table S10.

For the morphological traits, average measurements for all horses assessed at breeding field tests in 2019–2021, as recorded in the Worldfengur database, were used as a reference. The selection aimed at choosing elite horses that were measured equal to or higher at the front and with equal or less back inclination than the average assessed horse. Conversely, horses with lower front measurements and greater back inclination compared to the average assessed horses were classified as non-elite. However, three elite and three non-elite horses deviated from the measurement criteria by 1–2 cm for one or both traits. Additionally, four elite and two non-elite horses deviated from the test score criteria by 0.5-1.0 points for one, two or three traits. These deviations occurred primarily because DNA samples were more readily available from horses that met most, but not all, of the criteria. Furthermore, three horses had low pace scores (6.5-7.0) but received outstanding scores for tölt and slow tölt (9.0-9.5) and were therefore categorised as four-gaited elite horses. Nonetheless, elite horses had higher pace scores on average than non-elite horses. The characteristics of the selected horses in the two groups and descriptive statistics for the selection traits in each group are presented in Tables 4 and 5. Furthermore, the distribution of test scores and measurements for the selection traits in the two groups are shown with histograms in Supplementary Fig. S1.

**Table 4:** Descriptive characteristics of the horses included in the study, categorized into elite and non-elite groups. The table provides information on the total number of horses in each group, their biological sex, the number of four- and five-gaited horses, birth year, year of assessment at the breeding field test, age at the time of assessment, and the country where the assessment took place.

Characteristics	Elite horses (*N* = 22)	Non-elite horses (*N* = 17)
Sex		
*Stallions/geldings*	14	5
*Mares*	8	12
Performance type		
*Four-gaited*	13	5
*Five-gaited*	9	12
Birth year		
*Min*	2006	2006
*Max*	2017	2015
Assessment year		
*Min*	2012	2017
*Max*	2022	2023
Age when assessed		
*Min*	4	5
*Max*	11	14
*Mean*	6.4	8.9
*Standard deviation*	1.5	2.8
Country of assessment		
*Iceland*	21	6
*Sweden*	1	11
DMRT3 ‘gait keeper’ genotype		
*AA*	22	13
*CA*	0	4
*CC*	0	0

**Table 5:** Descriptive statistics for the selection traits in the elite and the non-elite horse groups. *Tölt*,* slow tölt*,* Pace* and *Back and croup* were subjectively assessed according to a judging scale from 5.0–10. The trait *Pace ≥ 5.0* includes all Pace scores, even the 5.0 score indicating that the trait was not shown at the test, while the trait *Pace > 5.0* only includes Pace scores when the trait was shown at the test. The morphological traits, *Height at front* and *Backline* were measured in centimetres; where *Height at front* is the difference between the highest point on withers (M1) and the tuber sacrale (M3), *Backline* is the difference between the height at the tuber sacrale (M3) and the lowest point of the back (M2).

	Elite horses				Non-elite horses			
Selection traits	Mean	Sd	Min	Max	Mean	Sd	Min	Max
Tölt	9.2	0.4	8.5	10.0	7.2	0.4	6.5	8.0
Slow tölt	8.8	0.6	7.5	10.0	7.1	0.5	6.0	7.5
Pace ≥ 5.0	6.9	1.9	5.0	9.5	6.2	0.9	5.0	7.5
Pace > 5.0	8.5	1.0	6.5	9.5	6.7	0.5	6.0	7.5
Back and croup	8.9	0.4	8.0	9.5	7.1	0.3	6.5	7.5
Height at front (M1-M3)	6.7	1.5	4.0	10.0	2.7	1.5	1.0	7.0
Backline (M3-M2)	4.9	1.4	2.0	8.0	7.6	2.0	4.0	10.0

### Sample collection and DNA preparation

Blood samples were collected in Sweden and Iceland according to ethical approvals. This study was approved by the Ethics Committee for Animal Experiments in Uppsala, Sweden (number: 5.8.18–15453/2017 and 5.8.18–05055/2019) and an animal experiment license by the Icelandic Food and Veterinary Authority in Iceland (number: 2020-04-02/2003120). The study was carried out in accordance with ARRIVE guidelines. The blood samples were collected from the selected Icelandic horses by certified veterinarians in accordance with relevant guidelines and regulations after acquiring informed consent from the horse owners. All the horses were privately owned and under the care of their owners. Genomic DNA from the blood samples was extracted using an automated DNA extraction machine, following a standard protocol. The concentration of all the DNA samples was measured using a spectrophotometer, with an OD260/280 ratio of at least 1.8–2.0. A quality check (DIN > 9.0) was made on random samples with an automated electrophoresis platform.

### Mapping and variant calling

The genomic DNA was sequenced on an S4 flowcell using the Illumina NovaSeq 6000 system with a 150 bp paired-end read length. FastQC software version 0.11.9 (bioinformatics.babraham.ac.uk/projects/fastqc/) was used to assess the quality of raw reads. Quality processing and trimming of the raw reads was done using Fastp v0.23.2^52^. The processed reads were mapped to the EquCab3.0 reference genome (Ensembl v110) using the bwa-mem program in Burrows-Wheeler Aligner^53^. Variants were called using Genome Analysis Toolkit (GATK) v4.5.0 following GATK best practices^54^. Briefly, PCR duplicate reads in the BAM files were flagged using MarkDuplicatesSpark, followed by base quality score recalibration of subsequent BAM files. Variants (SNPs and INDELs) were called for each sample using the GATK HaplotypeCaller with the “-ERC” flag^55^. This per-sample analysis generated intermediate files called genomic variant calling format (gVCF) that have a record for every genomic position. We combined the gVCF files using the GATK CombineGVCFs program and performed joint genotyping for high-confidence alleles using GenotypeGVCFs. To minimize the number of false positives, hard filtering of the joint files was performed using the GATK best practice guidelines^54^. The hard-filtered joint files were used for downstream analysis.

### Linkage disequilibrium estimates

Variants in linkage disequilibrium (LD) with the top SNPs that were previously shown to be associated with pace^7^ in SNP genotype data with lower density, were identified using the LD function in PLINK (version 1.9)^56,57^. The window size was set equal to 3 Mb in order to ensure the detection of all variants in LD. Only SNPs and indels with an r^2^ value greater than 0.8 were considered for further analysis. LD heatmap plots were generated using the LDheatmap package^58^ in R (v.4.3.3)^59^.

### Variant analyses

Variants comprising the two haplotypes were annotated and ranked using the Ensembl (release 112, May 2024)^60^ Variant Effect Predictor (VEP)^61^. The conservation of variants across taxa, such as Genomic Evolutionary Rate Profiling (GERP scores)^62^, was extracted using the Ensembl database. The JBrowse page for the Equine section of Functional Annotation of Animal Genomes (FAANG)^63^ was used to map the variants to known tissue-specific chromatin immunoprecipitation sequencing (ChIP-seq) peaks for histone modification marks^19,20^. Data was available from adipose, brain, heart, lamina, liver, lung, muscle, and ovary tissues. PhyloP scores were extracted from the UCSC Genome Browser^64^ by using the LiftOver tool^65^ to translate genomic coordinates from EquCab3.0 into the human (GRCh38/hg38) genomic coordinate identities. Multiple PhyloP scores based on different species sets^66^ and alignment algorithms^67,68^ were investigated (Vertebrate Multiz alignment and conservation considering 100 species; Cactus alignment and conservation of Zoonomia placental mammals considering 241 species; Cactus alignment and conservation on 447 mammal species, including Zoonomia genomes; Multiz alignment and conservation considering 470 mammals). The human coordinates were further used to investigate possible variant overlaps with known regulatory elements, such as transcription factor binding sites (TFBS), using the JASPAR CORE 2024^69^ track in the UCSC Genome Browser^64^ and to investigate possible variant associations with known expression quantitative trait loci (eQTL) and splicing quantitative trait loci (sQTL) using the Genotype-Tissue Expression (GTEx) portal^70^. Possible splice sites were also explored by importing a custom track generated with the SpliceAI-visual tool^71^ into the UCSC Genome Browser. Finally, the Expasy translate tool^72^ was used to translate coding sequences to protein sequences, and the Swiss-Model tool^73^ for protein modelling.

### Estimated phenotypic differences

The least squares means for groups of horses harbouring the same sets of haplotypes were assessed for breeding field test scores and measurements included in this study. This was done by using the glm() function for a general linear model in R (v.4.3.3)^59^. Analysis of variance (ANOVA) was used as a post-hoc test to identify significant phenotypic differences between those groups. The significance level was set at a *p*-value ≤ 0.05. Various fixed effects were tested in the model, including sex (male or female), age at assessment (categorized into four groups: 4, 5, 6, and ≥ 7 years old), age at assessment as a continuous covariate, and the time period of assessment (< 2020, ≥ 2020), as the judging scale for breeding assessments was revised in 2020^34^. The fixed effects of sex and age at assessment in years as a continuous covariate showed significant effects (*p* ≤ 0.05) for several traits. However, since the investigated genes in this study may influence learning behaviour, which could affect the age at which horses are ready for assessment, only the fixed effect of sex was included in the model for further analyses.

The fixed effects of assessment location (country) and the *DMRT3* genotype were not tested due to potential confounding. Nearly all elite horses were assessed in Iceland, while most non-elite horses were assessed in Sweden. Additionally, only four horses had the CA genotype for the *DMRT3* mutation, and all of these were non-elite mares.

### Allele frequencies

To compare allele frequencies for the variants of interest in Icelandic horses and other breeds, this study included publicly available genotype and sequence data from previous studies^25–31^ and the European Variation Archive^74^ (https://www.ebi.ac.uk/eva/) with accession numbers PRJEB47918 and PRJEB55177. No horses overlapped between studies. A list of the studied breeds, including the number of horses in the samples and the corresponding reference or data accession number, is provided in Supplementary Table S9.

## Electronic supplementary material

Below is the link to the electronic supplementary material.

Supplementary Material 1

Supplementary Material 2

## Data Availability

The sequence data generated and analysed during the current study are available in the European Variation Archive^74^ (project ID: PRJEB82503, https://www.ebi.ac.uk/eva/?eva-study=PRJEB82503). The phenotype data analysed during the current study are not publicly available due to commercial value for the Icelandic horse breeding industry. Still, they are available from the corresponding author upon reasonable request and with permission from the Icelandic Agricultural Advisory Center (RML) and the Swedish Icelandic Horse Association (SIF Avel).
