# Supplementary material for: Genetic influence of a STAU2 frameshift mutation and RELN regulatory elements on performance in Icelandic horses
Source: Sci Rep. 2025 Apr 4;15:11641. doi: 10.1038/s41598-025-95593-8 (PMC11971302; doi:10.1038/s41598-025-95593-8)
Supplement: Supplementary file 2 — Supplementary Material 2 [file 41598_2025_95593_MOESM2_ESM.pdf]

Genetic influence of a *STAU2* frameshift mutation and *RELN* regulatory elements on performance in Icelandic horses

Heiðrún Sigurðardóttir[1,2*], Susanne Eriksson[1], Adnan Niazi[1], Marie Rhodin[1], Elsa Albertsdóttir[3], Thorvaldur Kristjansson[2,4], and Gabriella Lindgren[1,5]

[1]Swedish University of Agricultural Sciences, Department of Animal Biosciences, P.O. Box 7023, SE-75007 Uppsala, Sweden,

[2]Agricultural University of Iceland, Faculty of Agricultural Sciences, Hvanneyri, IS-311 Borgarbyggð, Iceland,

[3]Independent researcher, IS-203 Kópavogur, Iceland

[4]The Icelandic Agricultural Advisory Centre, Höfðabakka 9, IS-110 Reykjavik, Iceland,

[5]KU Leuven, Center for Animal Breeding and Genetics, Department of Biosystems, Kasteelpark Arenberg 30, BE-3001 Leuven, Belgium

Distribution of test scores and body measurements for the selection traits in the two groups of elite and non-elite horses.

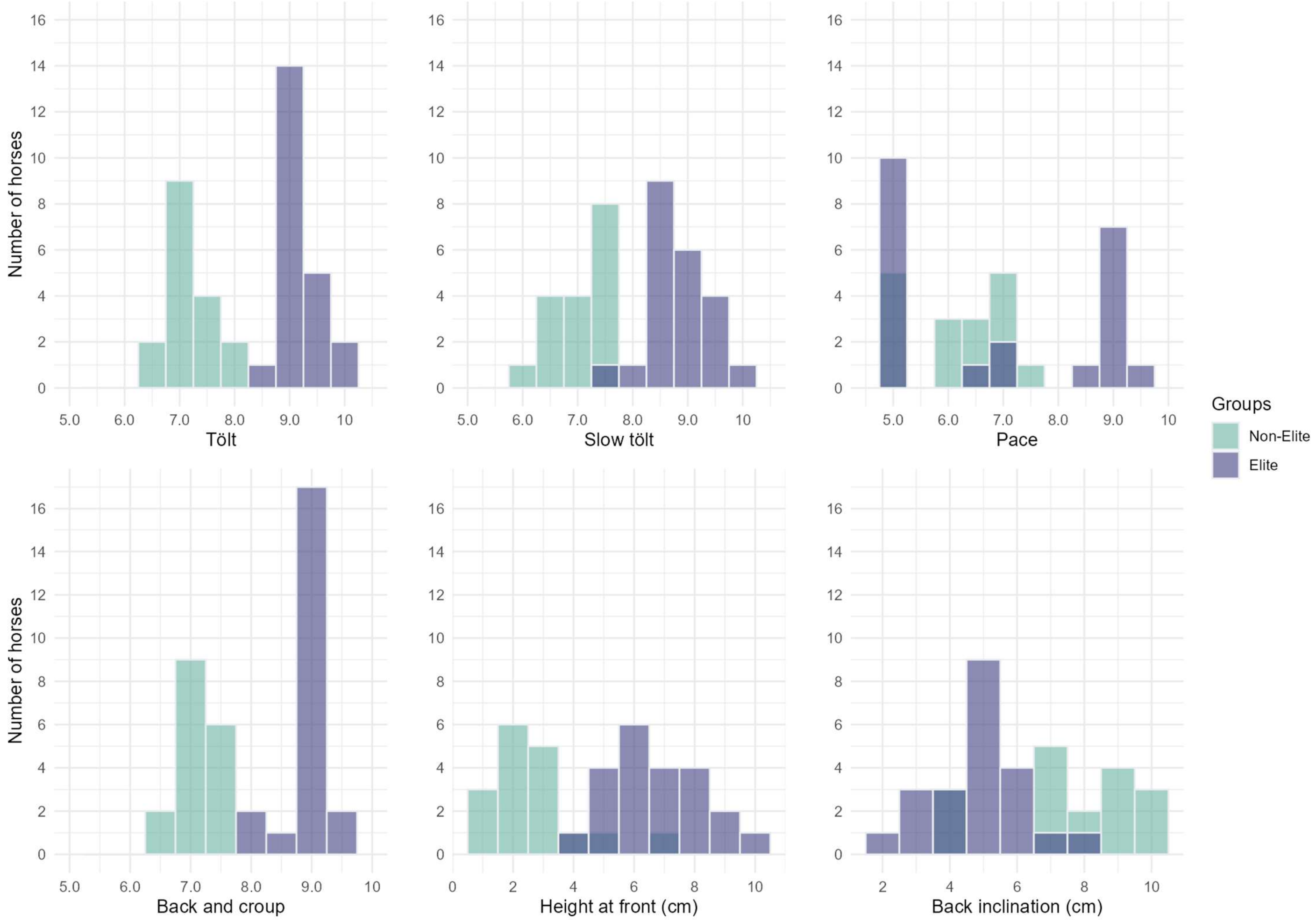

Figure S1: Distribution of test scores and body measurements for the selection traits in the two groups of elite and non-elite horses. The breeding field test scores are assessed on a scale from 5.0 to 10 with a 0.5 interval. The body measurements are recorded in centimetres: height at front is the difference between the highest point on withers (M1) and the tuber sacrale (M3), backline is the difference between the height at the tuber sacrale (M3) and the lowest point of the back (M2).
